# Effects of ultraviolet radiation on metabolic rate and fitness of *Aedes albopictus* and *Culex pipiens* mosquitoes

**DOI:** 10.7717/peerj.6133

**Published:** 2018-12-18

**Authors:** Oswaldo C. Villena, Bahram Momen, Joseph Sullivan, Paul T. Leisnham

**Affiliations:** 1 Marine Estuarine & Environmental Sciences, University of Maryland, College Park, MD, United States of America; 2 Department of Environmental Science & Technology, University of Maryland, College Park, MD, United States of America; 3 Department of Plant Science & Landscape Architecture, University of Maryland, College Park, MD, United States of America

**Keywords:** Metabolic rate, Microbial communities, UV-B radiation, Vector competence

## Abstract

Natural and anthropogenic changes (e.g., land use change, pollution) will alter many environmental factors in the coming years, including the amount of solar radiation reaching the earth’s surface. Alterations in solar radiation exposure is likely to impact the ecologies of many living organisms, including invertebrates that inhabit aquatic habitats. In this study, we assessed the effect of UV-B radiation on the metabolic rates and fitness (survival, development time, body size) of *Aedes albopictus* and *Culex pipiens* mosquitoes and the activity of their microbial food resources in experimental aquatic microcosms*.* We exposed single-species cohorts of newly hatched *Ae. albopictus* and *Cx. pipiens* larvae and a control treatment with no larvae to three UV-B conditions that mimicked those in full-sun and shade in the field and to a control condition with no UV-B radiation. Our results indicated that UV-B radiation affected the metabolic rates of both *Ae. albopictus* and *Cx. pipiens* larvae, with significantly higher rates found in full-sun compared to shade and no-UV conditions, 8 and 15 days after exposure began. *Ae. albopictus* and *Cx. pipiens* survival was also affected by UV-B radiation condition, with significantly lower survival in full-sun compared to shade and no UV-B conditions. Microbial metabolic rates were consistently significantly lower in full-sun compared to shade and no-UV conditions, especially at 8 days of exposure. These results show that UV-B radiation at levels found in open spaces showed strong and important impacts on the metabolic rates and survival of *Ae. albopictus* and *Cx. pipiens* larvae. Decreased survival of *Ae. albopictus* and *Cx. pipiens* with higher UV-B radiation levels may be caused by both direct exposure to radiation as well as the indirect effects of reduced microbial food, resulting in greater metabolic demands and stress. Negative impacts of UV-B radiation on the survival of *Ae. albopictus* and *Cx. pipiens* are likely to have important implications for the distribution and abundance of these mosquitoes, and the transmission of pathogens that these two broadly distributed mosquitoes vector.

## Introduction

Environmental changes (e.g., global warming, climate change) will trigger major changes in environmental factors (e.g., temperature, precipitation) in coming years (Macnaghten & Szerszynski, 2013; Bornman et al., 2015). These changes are likely to have profound impacts on insect ecology and physiology, including survival, development time, and underlying metabolic processes (Helbling & Zagarese, 2003; Shuman, 2011; Gray, 2013). Much of the literature on global change biology has so far focused on temperature, precipitation, and humidity due to their well-characterized effects on arthropods (Pedrosa de Almeida et al., 2010; Jaworski & Hilszczanski, 2013; Schulte, 2015; Sinclair et al., 2016). Other environmental changes, including increased exposure to ultraviolet radiation (UVR) from climate change (e.g., cloud thickness) and anthropogenic activities (e.g., land use change, pollution), may have subtler yet important effects that have so far been poorly studied.

UVR is part of the electromagnetic spectrum emitted by the sun, with a wavelength range between 100 and 400 nm (Andrady et al., 1998). UVR is subdivided into three subtypes by the length of the waves: UV-A, 315–400 nm; UV-B, 280–315 nm; UV-C, 100–280 nm. Only UV-A and UV-B radiation reach the earth’s surface (IARC, 2012), and UV-B radiation is considerably more harmful to living organisms than UV-A because of its shorter wavelength and consequently higher energy levels (Andrady et al., 1998). Although there is considerable variation in exposure to UV-B radiation throughout the landscape because of varying shade conditions, relatively few studies have examined the effects of UV-B radiation on insect behavior, physiology, and ecology (e.g., Johansen et al., 2011; Tuncbilek, Ercan & Canpolat, 2012; Shimoda & Honda, 2013; Sliney, Gilbert II & Lyon, 2016).

Most mosquito species feed on vertebrates for blood protein, and some species are medically important because they transmit pathogenic agents (e.g., parasites, viruses) that can cause disease (Darsie & Ward, 2016). *Aedes albopictus* (Skuse) (Diptera: Culicidae) and *Culex pipiens* (L.) (Diptera: Culicidae) are among the most common urban mosquitoes in the northeastern United States (Leisnham & Slaney, 2009; Costanzo, Mormann & Juliano, 2005). *Ae. albopictus* is an important vector for the transmission of many arthropod viruses, including yellow fever, dengue, and Chikungunya (Lambrechts, Scott & Gubler, 2010). *Ae. albopictus* is also capable of hosting the Zika virus, and it is therefore considered a potential vector in the field (Wong et al., 2013). *Cx. pipiens* is an important vector for the transmission of West Nile virus and Japanese encephalitis (Gerhardt et al., 2001; Kim et al., 2005; Molaei et al., 2006). *Ae. albopictus* and *Cx. pipiens* are also capable of transmitting dog heartworm (*Dirofilaria immitis*), which affects dogs, cats, foxes, coyotes, and other animals (Cancrini et al., 2007).

Mosquitoes oviposit eggs in aquatic habitats where the larvae and pupae typically develop within several weeks and then emerge into terrestrial adults (Clements, 1992). Mosquito larvae primarily feed on microbial organisms that colonize plant and animal detritus (Walker et al., 1991; Merritt, Dadd & Walker, 1992). Larval physiology and ecology affect the distribution and abundance of adults by moderating survival and adult fitness parameters, including such as body size, which can affect adult survival, biting rate, and ultimately the ability to vector and transmit pathogens (Terblanche & Chown, 2007). There is little information on the effect of UV-B radiation on the metabolic rates and survival of mosquitoes or on the microbial food resources on which they feed. To our knowledge only one study has rigorously assessed the effects of UVR on mosquitoes (MacGregor, 1932), which demonstrated clear negative effects of increasing UVR on larvae and pupae of *Ae. aegypti* (L.) and *Cx. pipiens*. However, a significant limitation of the study is that the UVR levels used in the study were not comparable to field conditions.

Other studies have demonstrated effects of UV-B radiation on microbial communities (e.g., Pancotto et al., 2003), but none have examined how these effects may impact mosquito populations. Future changes in UV-B radiation exposure from climate change and other anthropogenic activities (e.g., land use change, pollution) may have as important effects on microbial productivity as the underlying detritus on which the microbial organisms colonize (Ballare et al., 2011). The goal of this study is to test the effect of field-relevant UV-B radiation on the metabolic rates and fitness (survival, development time, and body size) of *Ae. albopictus* and *Cx. pipiens*, and on the production of the microbial communities on which they feed.

## Materials and Methods

### Collection and maintenance of mosquitoes

*Ae. albopictus* and *Cx. pipiens* larvae were collected from multiple locations in College Park, Baltimore, and Towson, Maryland (USA). Neither *Ae. albopictus* or *Cx. pipiens* are endangered and collection sites were either on publicly accessible lands or on private lands where consent for collections was granted at the time of collection; thus, no field permits were required to collect them. Field collected *Ae. albopictus* and *Cx. pipiens* larvae were reared to adulthood at 25 °C at 16:8 (L:D) h photoperiod and then released into 1-m^2^ single-species cages. Adults were kept in an insectary at 25 °C, >85% RH, and 16:8 (L:D) h photoperiod. Both colonies were supplied 20% sugar solution. Females from both colonies were fed horse or rooster blood once a week via an artificial feeder (Hemotek, Accrington, UK) to ensure egg production and experimental larvae. *Ae. albopictus* females oviposited on seed paper in 500 ml black cups covered filled with 200 ml of deionized (DI) water. Eggs were collected over multiple weeks and stored at >80% RH and 16:8 h (L:D) photoperiod until hatching for the experiment. *Cx. pipiens* oviposited egg rafts into a 500 ml black bowl filled with 400 ml of DI water. *Cx. pipiens* eggs cannot be held without hatching; thus, egg rafts were collected within 24 h of oviposition, hatched in a lactalbumin:yeast solution, and larvae were transferred into the experiment after being rinsed. *Ae. albopictus* eggs that had been stored were also hatched in a lactalbumin:yeast solution and transferred into the experiment after being rinsed and within 24 h of hatching. Experimental larvae of both species were *F*_1–3_ generation.

### Experiment set up

The experimental design was a split plot-randomized complete block design (RCBD) with UV-B radiation condition (full-sun, 7.66 kJ m^−2^ d^−1^; shade, 4.26 kJ m^−2^ d^−1^; no-UVR control group, 0 kJ m^−2^ d^−1^) as the main plot and species treatment (*Ae. albopictus*, *Cx. pipiens*, no larvae) as the sub-plots. Within each sub-plot there were five sub-samples consisting of 20 ml vials with 17 ml DI water and single-species mosquito cohorts of 10 newly-hatched *Ae. albopictus* or *Cx. pipiens* larvae, or no larvae. Each vial was inoculated with 1 ml of homogenized water from discarded field tires to allow microbial communities to establish. We ran three temporal replicates as random blocks. In each block, there were 15 vials of each species treatment to give 45 total vials per block, and 5 vials of each species treatment (15 total) were randomly allotted to one of three environmental chambers (Model I-36 VL; Percival Scientific Inc., Perry, IA, USA). Each chamber was assigned one of the three UV-B radiation conditions that mimicked levels of UV-B radiation typically measured in full-sun and shade conditions in the field, as well as a control condition with no UV-B radiation (Rinnan et al., 2005).

To achieve UV-B radiation levels for the full-sun and shade conditions, cellulose diacetate filters were applied to four UV-B-313 lamps (Q Panel Lab Products, Cleveland, OH, USA) in each chamber and vials were placed 5 cm and 20 cm from the lamps, respectively. For the no UV-B radiation treatment group, four regular 32-watt bulbs (Model 205047, Phillips, Eindhoven, Netherlands) were used to simulate a visible range of sunlight (400–700 nm). To assure accurate and uniform exposure to UV-B radiation, vials were rotated daily. A different UV-B radiation condition was applied to each chamber for each temporal block to minimize any confounding effects between chamber and UV-B radiation treatment. Each chamber was kept at 25 °C, 16:8 (L: D), and 80–90% RH to mimic typical summer conditions in the northeastern USA (Day et al., 1993; Li et al., 2006). Vials were checked daily and pupae collected into individual vessels until adult emergence. Adults were killed by drying (>24 h, 50 °C), and their wing lengths measured using a dissecting microscope and the image analysis system Image Pro Plus 6.0 (Media Cybernetics, Rockville, MD, USA). The experiment continued until all individuals had either died or emerged in all vials. Dead larvae were left in vials to mimic field conditions. For each vial, proportion survivorship to adulthood, mean development time, and mean wing length were calculated.

### Measurement of metabolic rates

Metabolic rates of both mosquito larvae and microbial communities were measured as the rate of heat production (µwatts/ml) by a heat conduction multicell differential scanning calorimeter (MC-DSC model 4100, Calorimetry Sciences Corp., Lindon, UT, USA) in isothermal mode and at 25 °C  ± 0.05, using sterile techniques and following procedures of past studies (Lighton, 2008; Zhang et al., 2009; Braissant et al., 2010). Measurements were made on days 1, 8, and 15, after adding larvae. On each measurement day, five live larvae were randomly sampled from each of the 30 vials containing mosquitoes, washed in sterilized water, and placed inside one of the two 1 cm^3^ testing ampoules with 1 ml of deionized water. Heat production was monitored for 60 min to allow for temporal equilibration and consistency of a final reading (Zhang et al., 2009; Braissant et al., 2010). To control for variation among individual ampoules, a baseline blank sample (deionized water only) was run immediately prior to every experimental sample. Its heat production was subtracted from that of the experimental sample from the same ampoule to yield a final metabolic rate value in µwatts/ml (Zhang et al., 2009). After each run, larvae were returned to their experimental vial. Ampoules were washed in sterilized water and ethanol between each run to avoid contamination (Gustafsson, 1991; Zhang et al., 2009; Braissant et al., 2010). Microbial metabolic rate was also measured from all 45 experimental vials by sampling 1 ml of vial water only and comparing it to a blank sample using the same procedure as described with larvae.

### Analyses

Metabolic rates of *Ae. albopictus*, *Cx. pipiens,* and microbial communities were analyzed as repeated measures three-way blocked analysis of variance (ANOVA). UV-B radiation condition, species treatment, and sampling day were treated as fixed effects, and chamber as a random block effect. Sampling day was a repeated factor within UV-B radiation condition, species treatment, and block. Mosquito fitness parameters were analyzed as two-way ANOVA, with UV-B radiation condition and species as fixed effects and chamber as a random block effect. To account for assumptions of normality and homogeneity of variances, metabolic rates and fitness parameters were log 10(*y*) and log 10(*y* + 1) transformed, respectively. All analyses were done using the PROC MIXED procedure, SAS 9.4 software (SAS Institute Inc., 2013). Multiple pairwise comparisons were conducted using the LSMEANS statement with Tukey adjustment. For all analyses, experiment-wise *α* = 0.05.

## Results

### Metabolic rates

**Figure 1:**
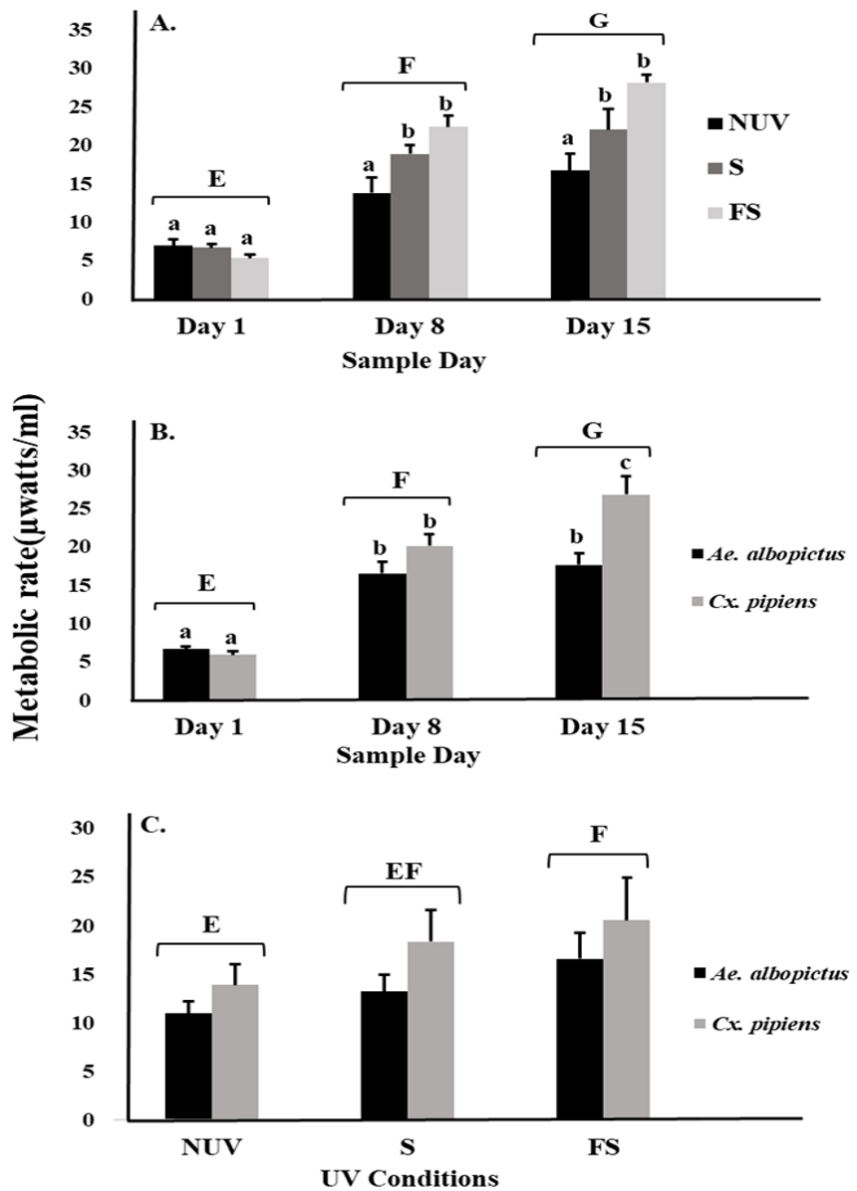
Metabolic rate of mosquitoes in response to UV-B radiation conditions and sample day. Least squares means (±SE) for metabolic rate expressed as heat production (µW/ml) of larvae of *Ae. albopictus* and *Cx. pipiens* in response to (A) interaction of sample day (days 1, 8, and 15) and UV-B conditions: no-UV (NUV), shade (S), and full-sun (FS), (B) day of metabolic rate measurement (days 1, 8, and 15), and (C) UV-B conditions (NUV, S, FS). Data were statistically tested using ANOVA. Significant pairwise comparisons among treatment levels for main effects of (A) sample day, (B) sample day, and (C) UV-B conditions are indicated by capitalized letters, and interaction effects of (A) sample day and UV-B conditions and (B) sample day and species are indicated by lowercase letters.

There was an effect on mosquito metabolic rates related to interactions between UV-B radiation condition and sample day (Table 1, Fig. 1A). The metabolic rates of both species increased over time to a peak on day 15 under full-sun and shade conditions but not under the no-UV condition (Fig. 1A). There was also an interaction between species and sample day, indicating differences in the metabolic rates of *Ae. albopictus* and *Cx. pipiens* depending on time (Table 1, Fig. 1B). On day 15, metabolic rates of *Cx. pipiens* were higher than for *Ae. albopictus* whereas this was not seen on days 1 and 8 (Fig. 1B). Main effects of UV-B radiation condition, species, and sample day were also detected (Table 1). Metabolic rates of both *Ae. albopictus* and *Cx. pipiens* were significantly higher under the full-sun condition compared to the no-UV condition and on days 8 and 15 compared to day 1 (Fig. 1C). Across all days and UV-B conditions, metabolic rates of *Cx. pipiens* were on average higher than *Ae. albopictus* (Figs. 1B and 1C).

**Table 1:** Analysis of variance of the effects of UV-B radiation conditions and species on mosquito larvae metabolic rates. Three-way ANOVA of the effects of UV-B conditions (full-sun, shade, and no-UV) and species (*Ae. albopictus* and *Cx. pipiens*) at three different times (days 1,8, and 15) on the larvae metabolic rate of *Ae. albopictus* and *Cx. pipiens* mosquitoes.

**Variable**	**Larval metabolic rate**		
	**dfs**	***F***	***P***
UV-B conditions	2,10	5.50	**0.0245**
Species	2,10	6.08	**0.0333**
UV-B conditions × species	2,10	0.58	0.5799
Sample day	2,24	350.85	**<0.0001**
UV-B conditions × sample day	2,24	13.96	**<0.0001**
Species × sample day	2,24	14.79	**<0.0001**
UV-B conditions × species × sample day	2,24	0.87	0.4975

**Figure 2:**
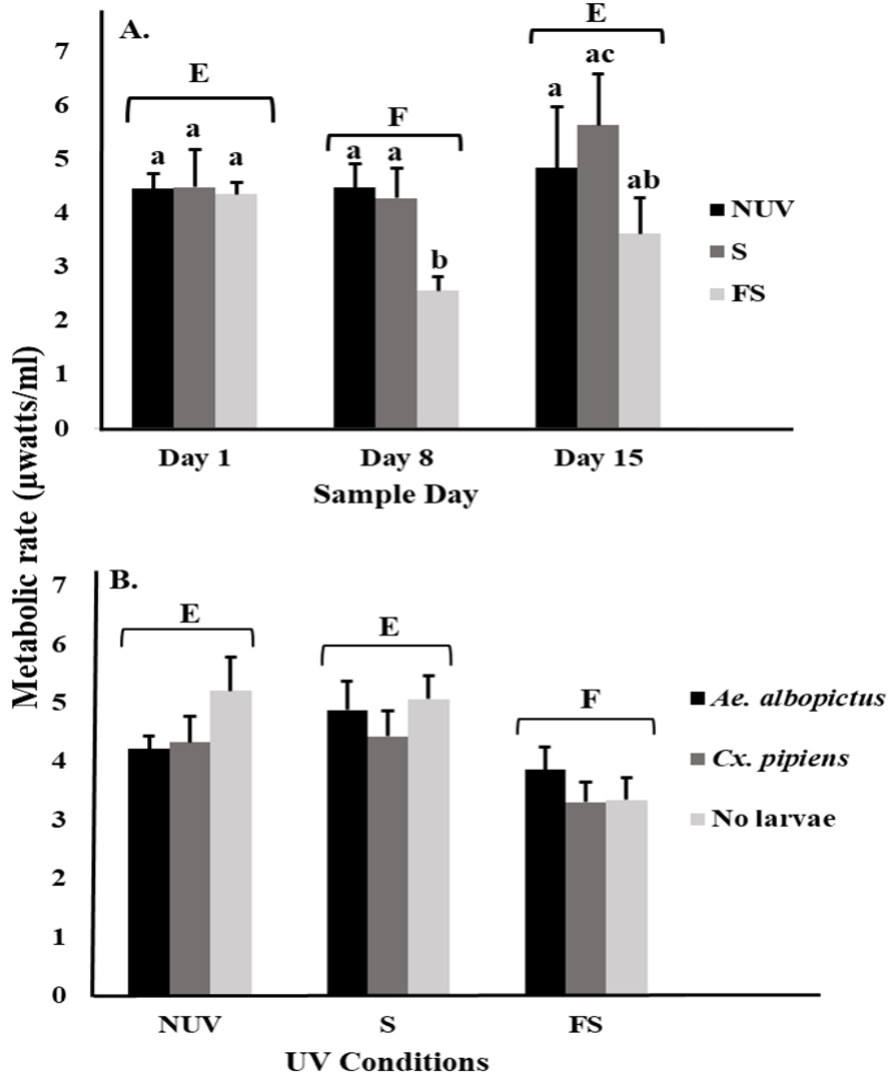
Metabolic rate of microbial communities in response to container inhabitants, UV-B radiation conditions, and sample day. Least squares means (±SE) for metabolic rate expressed as heat production (µW/ml) of microbial community from vials that contain *Ae. albopictus* larvae, *Cx. pipiens* larvae, and no larvae (just microbial community) in response to (A) interaction of sample day (days 1, 8, and 15) and UV-B conditions (NUV, S, and FS) and (B) main effects of UV-B conditions. Data were statistically tested using ANOVA. Significant pairwise comparisons among treatment levels for main effects of (A) sample day and (B) UV-B conditions are indicated by capitalized letters, and interaction effects are indicated by lowercase letters.

For microbial metabolic rates, there was an interaction between UV-B radiation condition and sample day (Table 2), with decreasing metabolic rates from day 1 to 8 under the full-sun condition only (Fig. 2A). This decrease appeared to drive the significant main effects of sample day and UV-B radiation condition (Table 2). Microbial metabolic rates were lower on day 8 compared to days 1 and 15 (Fig. 2A), and lower under the full-sun conditions compared to shade and no-UV conditions (Fig. 2B). Microbial metabolic rates did not vary between vials with *Ae. albopictus, Cx. pipiens,* or no larvae.

### Mosquito fitness

**Figure 3:**
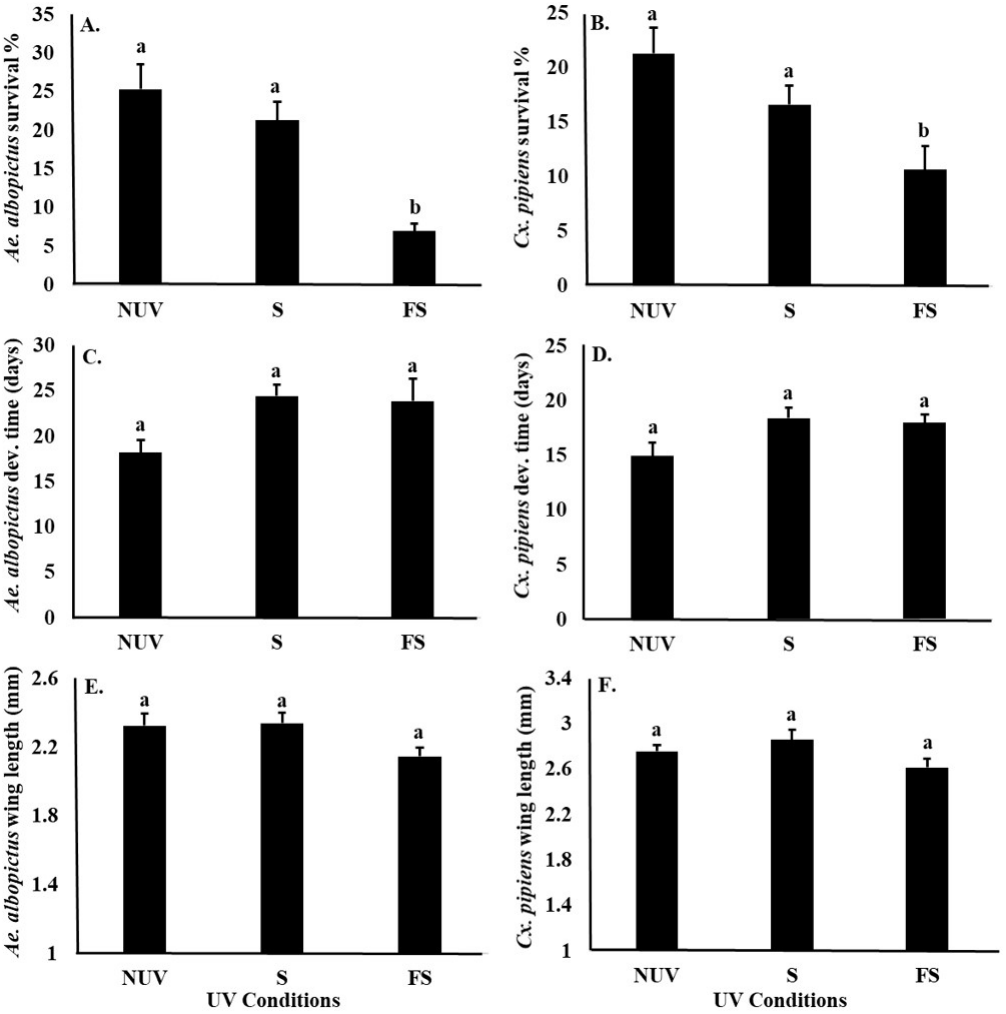
Mosquito fitness parameters in response to UV-B radiation conditions. Least squares means (±SE) for fitness parameters of *Ae. albopictus* and *Cx. pipiens* mosquitoes. (A) survival percentage of *Ae. albopictus,* (B) survival percentage of *Cx. pipiens,* (C) development time of *Ae. albopictus,* (D) development time of *Cx. pipiens,* (E) body size of *Ae. albopictus*, and (F) body size of *Cx. pipiens* in response to UV-B conditions: full-sun (FS), shade (S), and no-UV (NUV). Data were statistically tested using ANOVA. Significant pairwise comparison among treatment levels for main effects are indicated by different letters above bars.

UV-B radiation conditions affected the survival of *Ae. albopictus* and *Cx. pipiens* larvae similarly (Table 3), with significantly lower survival of both species under the full-sun condition compared to shade and no-UV conditions (Figs. 3A and 3B). There was also a main effect of species on female body size, with *Cx. pipiens* being on average larger than *Ae. albopictus* (Figs. 3E and 3F). There were no effects of UV-B radiation conditions or species on the development times of either *Ae. albopictus* of *Cx. pipiens,* or an interaction effect between UV-B radiation conditions and species on any fitness parameter (Table 3, Fig. 3).

## Discussion

Ultraviolet radiation (UVR), especially UV-B radiation, could have important effects on the distribution and abundance of pathogen-transmitting species. This is the first study that has rigorously tested the effects of UV-B radiation on the metabolic rates and fitness of medically important mosquitoes and the activity of their microbial food resources*.* Our results showed that UV-B radiation increased the metabolic rates of both *Ae. albopictus* and *Cx. pipiens* larvae, with significantly higher rates in full-sun conditions compared to shade conditions and a no-UV control after 8 and 15 days of exposure. In field conditions, metabolic rate increased in mosquito larvae between emergence and day 4–5 (Gray & Bradley, 2003). Microbial metabolic rates were also lower in full-sun conditions after 8 days of exposure compared to shade and no-UV conditions. These results suggest that UV-B radiation at levels typically found in open spaces in the field is likely have strong and important impacts on the ecologies of *Ae. albopictus* and *Cx. pipiens* mosquitoes and potential disease transmission.

**Table 2:** Analysis of variance of the effects of UV-B conditions, species inhabiting vials, and time on the metabolic rates of microbial communities. Three-way ANOVA of the effects of UV-B conditions (full-sun, shade, and no-UV) and the species that inhabit the vials where microbial samples come from (*Ae. albopictus, Cx. pipiens*, and no larvae) at three diferent times (days 1, 8, and 15) in the metabolic rate of microbial community.

**Variable**	**Microbial metabolic rate**		
	**dfs**	***F***	***P***
UV-B conditions	2,16	10.74	**0.0011**
Species	2,16	1.13	0.3483
UV-B conditions × species	4,16	0.79	0.5502
Sample day	2,36	5.69	**0.0071**
UV-B conditions × sample day	4,36	3.65	**0.0135**
Species × sample day	4,36	0.47	0.7562
UV-B conditions × species × sample day	8,36	0.54	0.8203

Higher metabolic rates for mosquito larvae under full-sun conditions, especially with increasing time of exposure, are likely to indicate increased energy expenditure to accomplish basic biologic processes, including feeding, development, and immunological functioning. Given that we saw commensurable decreases in larval survival and microbial activity, it is likely that larvae need to expend more energy to utilize declining food resources under full-sun conditions. Our findings are broadly consistent with those of other studies that have found simple and complex impacts of UV-B radiation on aquatic ecosystems (see Hader et al., 2007 and references therein). For example, UV-B radiation can penetrate up to 3 m in clear freshwater habitats that have dissolved organic carbon (DOC) concentrations between 2 and 5 mg L^−1^, resulting in severe reductions in biomass of major producers (e.g., phytoplankton) and consumers (e.g., zooplankton, fish) within the food web (e.g., Williamson & Zagarese, 2003). Davidson & Belbin (2002) found that marine phytoplankton concentration and biomass decreased in 40% and 60% respectively in habitats less than 2 m deep when exposed to UV-B radiation for more than a day.

In this study, survival was the only mosquito fitness parameter negatively affected by UV-B radiation condition. Environmental stresses on insects are often manifested and detected through the release of hormones, such as cortisol, epinephrine, and octopamine (Peric-Mataruga, Nenadovic & Ivanovic, 2006; Farooqui, 2012). Releases of stress hormones as a result of UV-B radiation have also been associated with reductions in survival in at least one other insect (e.g., red flour beetle, *Tribolium castaneum*, Sang et al., 2016). Hormones also have key effects upon insect developmental time and body size (McBrayer et al., 2007). For example, the Prothoracicotropic hormone plays an important role in the release of ecdysone, a steroid hormone that affects the larval development time and body size in many insects, including mosquitoes (Zhang & Denlinger, 2011). UV-B radiation has been also been shown to delay the metamorphosis of *Tribolium castaneum* by influencing Prothoracicotropic hormone and ecdysteroid metabolism (Sang et al., 2016). Although we did not observe effects of UV-B radiation on the development time or body size of *Ae. albopictus* or *Cx. pipiens* in this study, it is possible that disruptions to the ecdysteroid metabolism in these and other mosquito species are mainly manifested in reduced survival.

**Table 3:** Analysis of variance of the effects of UV-B conditions and specie on the fitness parameters of mosquitoes. Two-way ANOVA of the effects of UV-B conditions (full-sun, shade, and no-UV) and specie (*Ae. albopictus* and *Cx. pipiens*) on the fitness parameters (survival, developmental time, and body size) of *Ae. albopictus* and *Cx. pipiens* mosquitoes.

**Variable**	**Survival**			**Development time**			**Body size-wing length**		
	**dfs**	***F***	***P***	**dfs**	***F***	***P***	**dfs**	***F***	***P***
UV-B conditions	2,11	7.11	**0.0104**	2,11	0.80	0.4773	2,11	1.05	0.3857
Species	1,11	0.01	0.9963	1,11	1.50	0.2491	1,11	16.36	**0.0023**
UV-B conditions × species	2,11	0.59	0.5717	2,11	0.30	0.7456	2,11	0.05	0.9518

Ecological theory and empirical evidence suggest that trade-offs occur among life-history traits when limited energy is partitioned among growth, maintenance, and reproduction (Bell, 1980). Environmental factors at the larval stage have been shown to affect a range of traits in adult mosquitoes, mostly via changes in body size, including susceptibility to viral infection (Alto et al., 2005), reproduction (Steinwascher, 1982), and longevity (Haramis, 1985; Reiskind & Lounibos, 2009), all of which can mediate changes in vectorial capacity (Araujo, Gil & e-Silva, 2012; Moller-Jacobs, Murdock & Thomas, 2014; Dickson et al., 2017). In addition to affecting larval survival, it is also possible that the effects of UV-B radiation at the larval life-stage might also manifest in adult traits (Dickson et al., 2017).

In addition to affecting mosquito populations through changes in microbial communities, we cannot disregard direct negative effects of elevated UV-B radiation on larvae. The only prior study to rigorously study the effects of UVR on mosquito larvae observed severe negative impacts on larval mobility (e.g., loss of movement coordination) and eclosion rates after exposure to UVR for 48 h (MacGregor, 1932). Histological analyses showed damage in the larvae cuticle, disintegration of the abdominal segments, and partial paralysis (MacGregor, 1932). However, MacGregor (1932) examined the effects of UV-C radiation (180–254 nm), which does not naturally reach the earth’s surface and is only commonly found from artificial sources unlikely to affect mosquitoes (e.g., mercury lamps, welding torches). To our knowledge, our study is the first to test field-relevant UVR on the ecology of mosquitoes and the activity of their microbial food resources.

The specific effects of UV-B radiation on the cells of living organisms is still unclear (Pelz-Stelinski, Kaufman & Walker, 2011). Prior studies have demonstrated that UV-B radiation damages the DNA, proteins, membranes, and photochemical efficiency of photosynthetic prokaryote organisms, affecting their photosynthesis and biomass production (e.g., Hader et al., 2007; Gao et al., 2008; Wu, Gao & Wu, 2009). For example, the spiral structure of cyanobacteria, *Arthrospira platensis*, can be broken and its photosynthetic activity disrupted with exposure to UV-B radiation within the temperature range of 18–20 °C, resulting in lower biomass (Gao et al., 2008). Wu et al. (2005) reported that exposure to 6 h of UV-B radiation broke the spiral filaments of *A. platensis* into small pieces, affecting its photosynthesis activity*,* as well as important photosynthetic electron transport and pigment-protein complexes.

Deforestation and the conversion of natural environments (e.g., grassland, agriculture) to build has increased over the last 100 years (Weng, 2010; Seto et al., 2011; Al-mulali et al., 2013). These land use changes are most likely to increase open spaces with full-sun conditions, although large building structures may increase shade in some areas (Wai, Yu & Lam, 2015). Seto, Guneralp & Hutyra (2012) predicts that by 2030 urban land cover will increase by 1.2 million km^2^ if current population trends continue. Urbanization also contributes to increasing pollutants from industrialization, vehicles, and electrical power generation (An et al., 2008; Al-mulali et al., 2013), many of which (e.g., CO_2_, smog, particulate matter) may absorb and reflect UVR away from the earth’s surface (Czerwinska et al., 2016). The combination of more open spaces and higher pollution could have complex impacts on the amount of UVR reaching ground surfaces in both space and time (Mandronich & Flocke, 1997).

*Ae. albopictus* and *Cx. pipiens*, two of the most broadly distributed mosquito species worldwide, are medically important because they are vectors for a number of arboviruses and they inhabit peridomestic areas (Costanzo, Mormann & Juliano, 2005; Leisnham & Slaney, 2009). West Nile virus (WNV) is the most important mosquito-borne human disease in the United States, with over 48,183 reported cases since being first detected in North America in 1999 (CDC, 2017). In the northeastern part of the United States, WNV is primarily circulated by *Cx. pipiens* among bird populations that amplify the virus (Kilpatrick, LaDeau & Marra, 2007). *Ae. albopictus* is capable of acting as a vector of WNV in the laboratory, but only rarely bites birds in the field and instead feeds on a range of hosts, including humans (Sardelis et al., 2002).

Anthropogenic environmental changes and the resultant increases in the exposure to UV-B radiation to container habitats could alter the distributions and abundances of these species. But their effects are difficult to predict and tease apart from associated changes to physio-chemical and biological conditions, including temperature, hydroperiod, and nutrients. Nevertheless, numerous studies have shown that *Cx. pipiens* is more likely to utilize containers that are more exposed to sunlight than *Ae. albopictus* and other mosquito species (Vezzani & Albicocco, 2009; Dowling et al., 2013); thus, we might expect *Cx. pipiens* to be especially prone to changes in UV-B exposure. On the other hand, *Ae. Albopictus*, which shows a strong preference to oviposit in shaded areas and at ground level (Amerasinghe & Alagoda, 1984; Williges, Faraji & Gaugler, 2014), could experience changes in its behavior, abundance, and distribution as responses to changes in UV-B exposure, changes that are likely to have important implications for the transmission of vector-borne diseases. It has been found that *Ae. albopictus* can occasionally lay eggs at greater heights depending on environmental factors like temperature (Toma et al., 2003) and habitat availability (Obenauer et al., 2009). For example, *Ae. albopictus* abundance could increase in dense urban areas (high density housing with high buildings) compared to peri urban/suburban areas (low density housing with low buildings), because of more abundant shaded environments to lay eggs, which could increase the risk of viral diseases spread by these mosquitoes. Li et al. (2014) found higher populations of *Ae. albopictus* in dense urban areas compared to peri urban areas in Guangzhou, China. *Ae. albopictus* also showed faster larval development, higher adult emergence rate, and longer survival time compared to peri urban and rural areas (Li et al., 2014), which could increase vector capacity. *Ae. albopictus* had showed high plasticity to adapt to new environments (Vitek & Livdahl, 2009; Tabbabi, 2018). Liew & Curtis (2004) showed that *Ae. albopictus* could vertically move as high as 60 m to oviposit in apartment buildings when there is not suitable habitat at ground level.

Better understanding of impacts of UV-B in the ecology of *Ae. albopictus* and *Cx. pipiens* is necessary to optimally assess human risk. By manipulating only UV-B radiation in a controlled laboratory experiment, our study was able to pinpoint the effects of UV-B radiation and demonstrated important impacts of altered exposure that we might expect from changes to shade.

## Conclusions

In summary, this study shows that UV-B radiation can have strong negative effects on the larval survival of both *Ae. albopictus* and *Cx. pipiens.* These effects were likely indirect, through decreases in the availability of microbial food, but could have also been direct, through adverse impacts on the cells of larvae. This is among the first studies to rigorously test the effects of UV-B radiation on the survival and fitness of mosquito larvae using levels typically observed in open and shaded habitats in the field. The impacts of UV-B radiation on larval survival are likely to affect the distribution and abundances of *Ae. albopictus* and *Cx. pipiens* but in ways that may be difficult to detect in the field since most landscapes are a patchwork of open and shaded areas that adult individuals can move between. Future research should examine the effects of UV-B radiation on the ecology, physiology, and behavior of other mosquito species and life-stages (eggs, adults), as well as on other community processes, such as predation, parasitism, and on vector competence across other disease systems such as chikungunya and Zika virus to gather a greater understanding of the potentially important impacts of UV-B radiation on vector ecology.

##  Supplemental Information

10.7717/peerj.6133/supp-1 Supplemental Information 1 Larvae metabolic rate

10.7717/peerj.6133/supp-2 Supplemental Information 2 Mosquito survival data

10.7717/peerj.6133/supp-3 Supplemental Information 3 Microbial metabolic rate

## Additional Information and Declarations

### Data Availability

The following information was supplied regarding data availability: Raw data are provided in the Supplemental Files.
